# Radio‐induced simultaneous development of squamous cell carcinoma and undifferentiated pleomorphic sarcoma of scalp

**DOI:** 10.1002/ccr3.6058

**Published:** 2022-07-14

**Authors:** Zahra Malakoutikhah, Fatemeh Mohaghegh, Maryam Derakhshan, Mohammad Javad Mehdizadeh

**Affiliations:** ^1^ Applied Physiology Research Center, Cardiovascular Research Institute Isfahan University of Medical Sciences Isfahan Iran; ^2^ Department of Dermatology, Skin Disease and Leishmaniasis Research Center Isfahan Medical School, Isfahan University of Medical Sciences Isfahan Iran; ^3^ Department of Pathology Isfahan Medical School, Isfahan University of Medical Sciences Isfahan Iran; ^4^ Department of Radiology Zahraye‐Marzieh Hospital Isfahan Iran

**Keywords:** pleomorphic sarcoma, radiodermatitis, radiotherapy, SCC

## Abstract

Radiotherapy was commonly applied to treat benign skin diseases such as tinea capitis. Some patients treated with radiation have developed skin malignancies over the years. This article reported a 72‐year‐old man presenting with two distinct non‐melanoma skin tumors on his scalp, including squamous cell carcinoma and metastatic pleomorphic sarcoma.

## Graphical abstract

The figure shows uneven scalp thickening over the left posterolateral aspect of the posterior fossa, associated with bone destruction of the medial aspect of the left occiput with intracranial extra‐axial extension representing a malignant process including sarcoma.

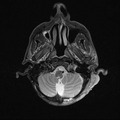

## 1 INTRODUCTION

From the 1930s to the 1960s, radiotherapy was applied widely to treat benign skin diseases like tinea capitis. The emergence of skin cancers is one of the most serious late side effects of radiation. Many studies have reported the induction of malignant diseases resulting from radiotherapy.^1^, ^2^ Notably, a fourfold increase in non‐melanoma skin cancers in the scalp has been reported among patients irradiated for tinea capitis. Hence, the risk of malignant transformation is significantly higher in radiodermatitis, estimated to be approximately 10%–20%. Surprisingly, the subsequent cancer is more aggressive than patients with skin cancers without radiation history.^3^

Basal cell carcinoma (BCC) is the most prevalent radio‐induced scalp cancer. However, squamous cell carcinoma (SCC) is frequently described as a result of radiotherapy for tinea capitis, with a mean latency period of 20 years. Radiation‐induced soft tissue sarcoma (RIS) is less common, representing a small proportion of soft tissue sarcomas, with a mean latency period of 11 years.^2^ The most prevalently reported RIS is undifferentiated pleomorphic sarcoma.^4^ This paper describes a rare case of SCC and metastatic undifferentiated pleomorphic sarcoma in the scalp of a patient who had previously had radiation for tinea capitis.

## 2 CASE PRESENTATION

**FIGURE 1:**
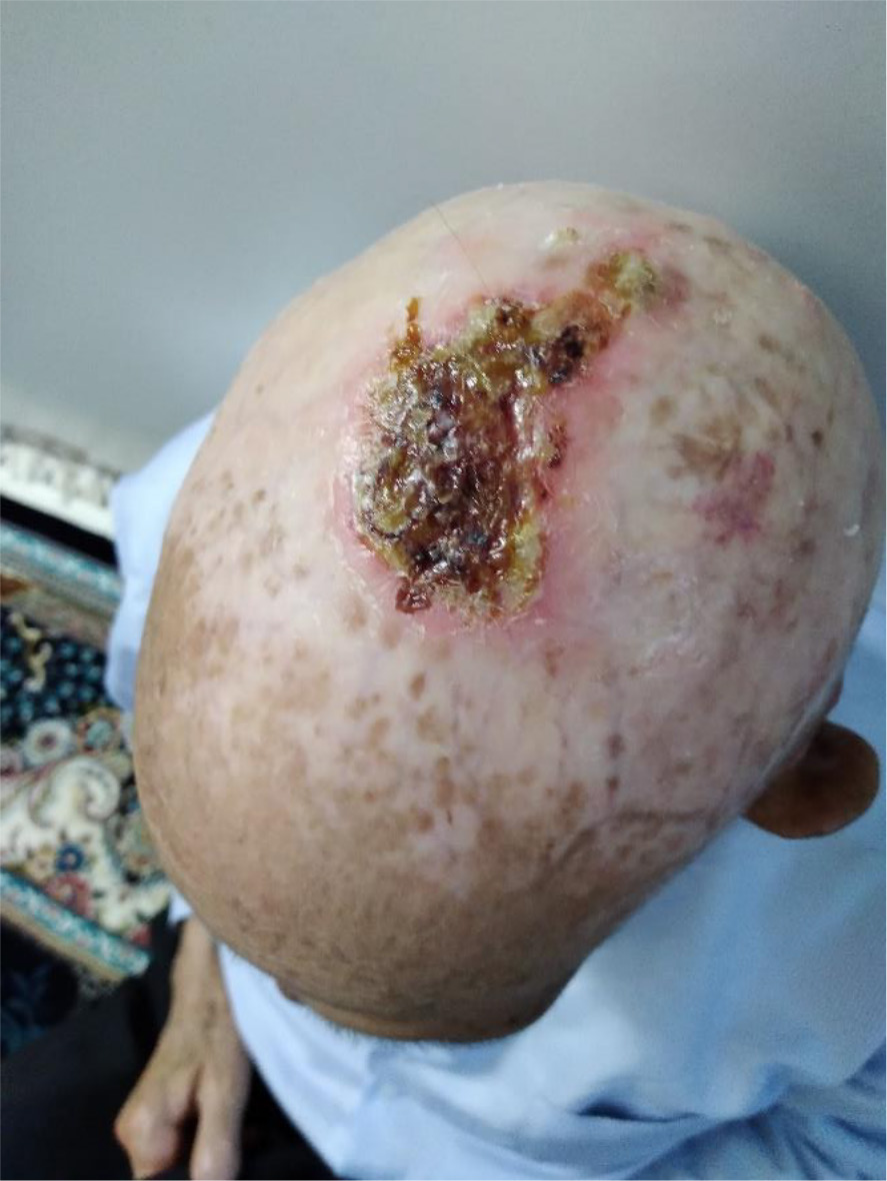
A 30 × 30 mm ulcerative lesion on the vertex with an SCC diagnosis

**FIGURE 2:**
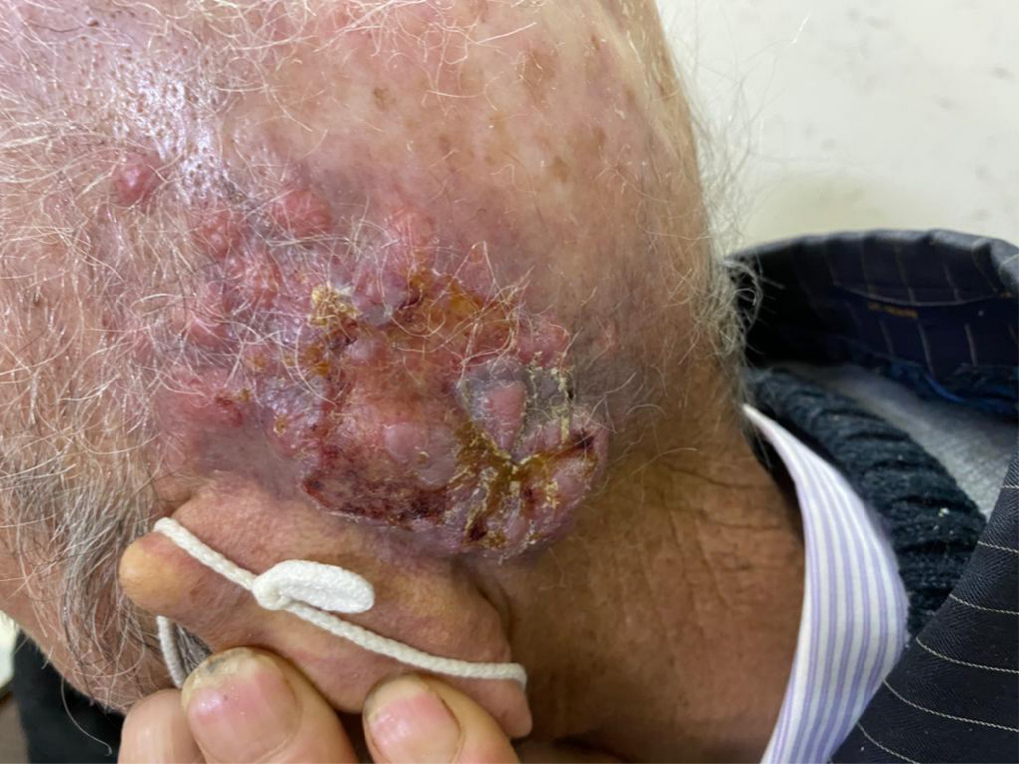
A 40 × 70 mm ulcerative lesion surrounded by infiltrative nodules in the left post‐auricular area of the scalp

A 72‐year‐old farmer man has been referred to our dermatology clinic for his scalp lesions. He presented with two lesions, one of which was an ulcerative lesion on the vertex with a size of 30 × 30 mm (Figure 1), and the other was an ulcerative lesion surrounded by the infiltrative nodules in the left post‐auricular part of the scalp with a size of 40 × 70 mm (Figure 2). However, the primary post‐auricular lesion was a small nodule that rapidly enlarged in 2 months, and he was referred to the dermatology clinic for further evaluation. He has been suffering from severe chronic radiodermatitis due to two radiotherapy sessions to treat tinea capitis in childhood. He had no other past medical and surgical history. He had no history of smoking, drugs, or alcohol. His brother had a lymphoproliferative disorder, and the incidence of different malignancies in his family was indicated.

**FIGURE 3:**
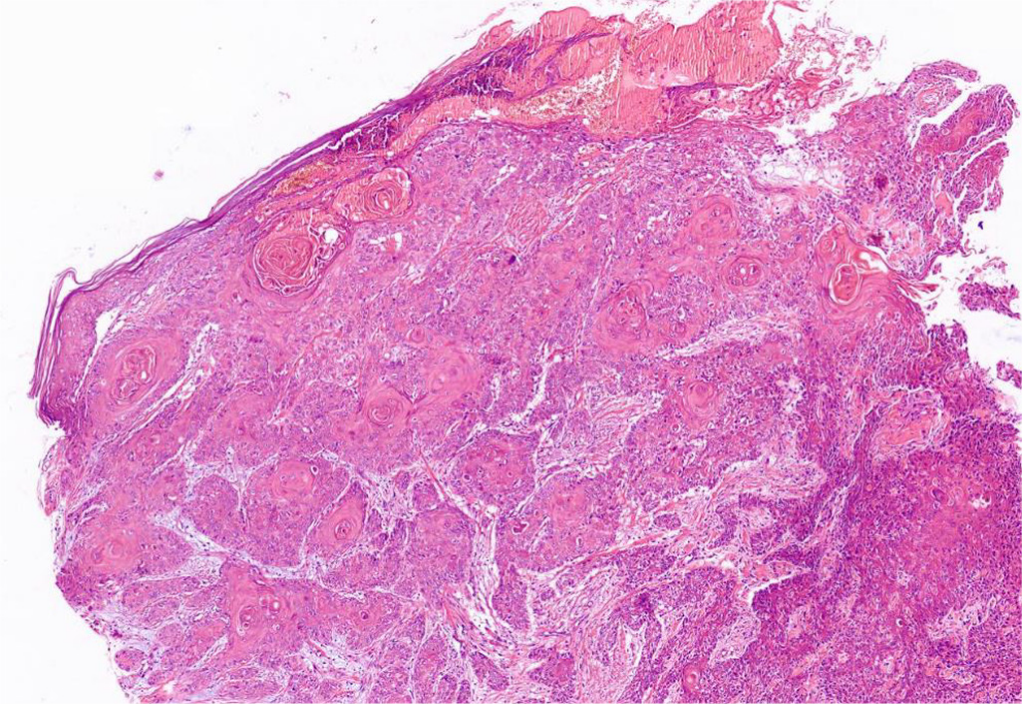
Scalp lesion, well‐differentiated squamous cell carcinoma.

**FIGURE 4:**
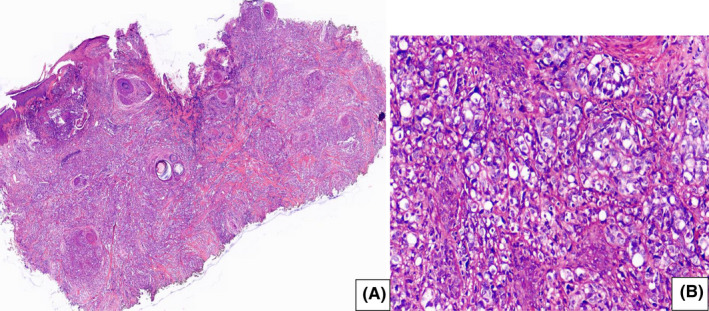
Post‐auricular lesion, (A) Ulcerated epithelium with diffuse infiltration of spindle and some pleomorphic neoplastic cells in the dermis, (B) High power, neoplastic cells have vesiculated nuclei with pleomorphism

The incisional biopsy was taken from both lesions. The vertex lesion had a neoplastic proliferation of squamous cells as a nested pattern with mild pleomorphism. The cell had a high n/c ratio, vesiculated nuclei, and eosinophilic cytoplasm. Keratin pearl was found in the background of tumoral cells. Invasion of the dermis was observed as well. The final diagnosis was well‐differentiated SCC (Figure 3). In the post‐auricular lesion, the epidermis was ulcerated and bizarre. In some areas, spindle neoplastic cells with vesicular nuclei, minute nucleoli, and abundant mitotic features were seen (Figure 4). According to the presence of well‐differentiated SCC in the vertex, poorly differentiated or spindle SCC was the first diagnosis. However, in the IHC staining, epithelial markers including CK, CK7, CK20, and specific markers for SCC (P63, CK5/6) all were negative.

**FIGURE 5:**
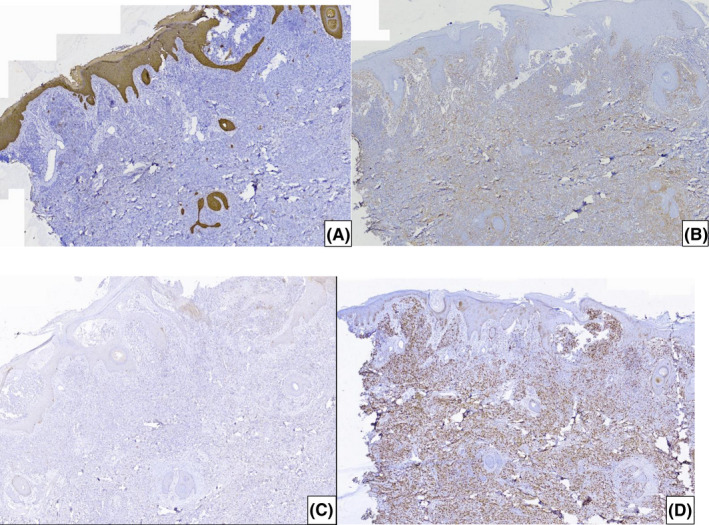
H/E staining IHC (A) CK is negative with internal control, (B) Vimentin is diffusely positive, (C) Melan A is Negative, and (D) Ki67 is positive (80–90% in tumoral cells)

The structure of the lesion in the H/E staining was poorly differentiated. Hence, it is suggested that IHC staining be performed to rule out malignant melanoma, lymphoma, specific sarcomas such as leiomyosarcoma and liposarcoma, as well as large cell neuroendocrine tumors. However, all markers, including SMA, CD34, Melan A, chromogranin, synaptophysin, and LCA, were negative. Only Vimentin was the positive marker, and the Ki67 index was about 80–90%, so high‐grade undifferentiated sarcoma was suggested (Figure 5).

Ultrasound showed pathological lymphadenopathy in the left superior jugular chain with a size of 13 × 22 mm, and in the right superior jugular chain with an extent of 18 × 30 mm. Conglomerated lymph nodes have been observed in the submandibular region and adjacent to the superficial lobe of the right parotid in a dimension of 14 × 40 mm. Moreover, other pathologic lymph nodes were seen in the left pre‐auricular region. The image of a soft tissue density, mainly hyperechoic and heterogenic, measuring 40 × 70 mm, was seen in the left post‐auricular area.

**FIGURE 6:**
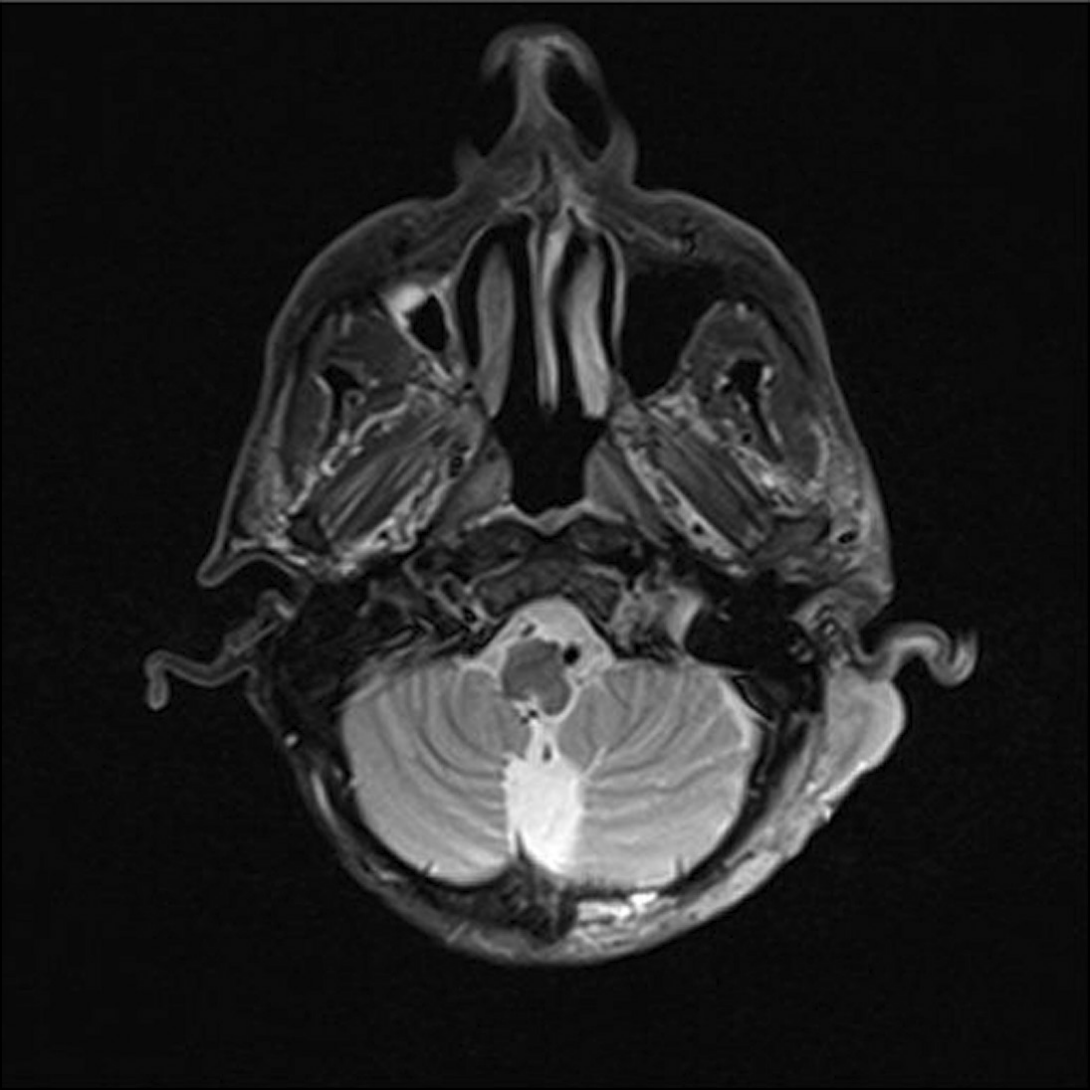
Axial T2W brain MRI shows uneven scalp thickening over the left posterolateral aspect of the posterior fossa, associated with bone destruction of the medial aspect of the left occiput with intracranial extra‐axial extension representing a malignant process including sarcoma

**FIGURE 7:**
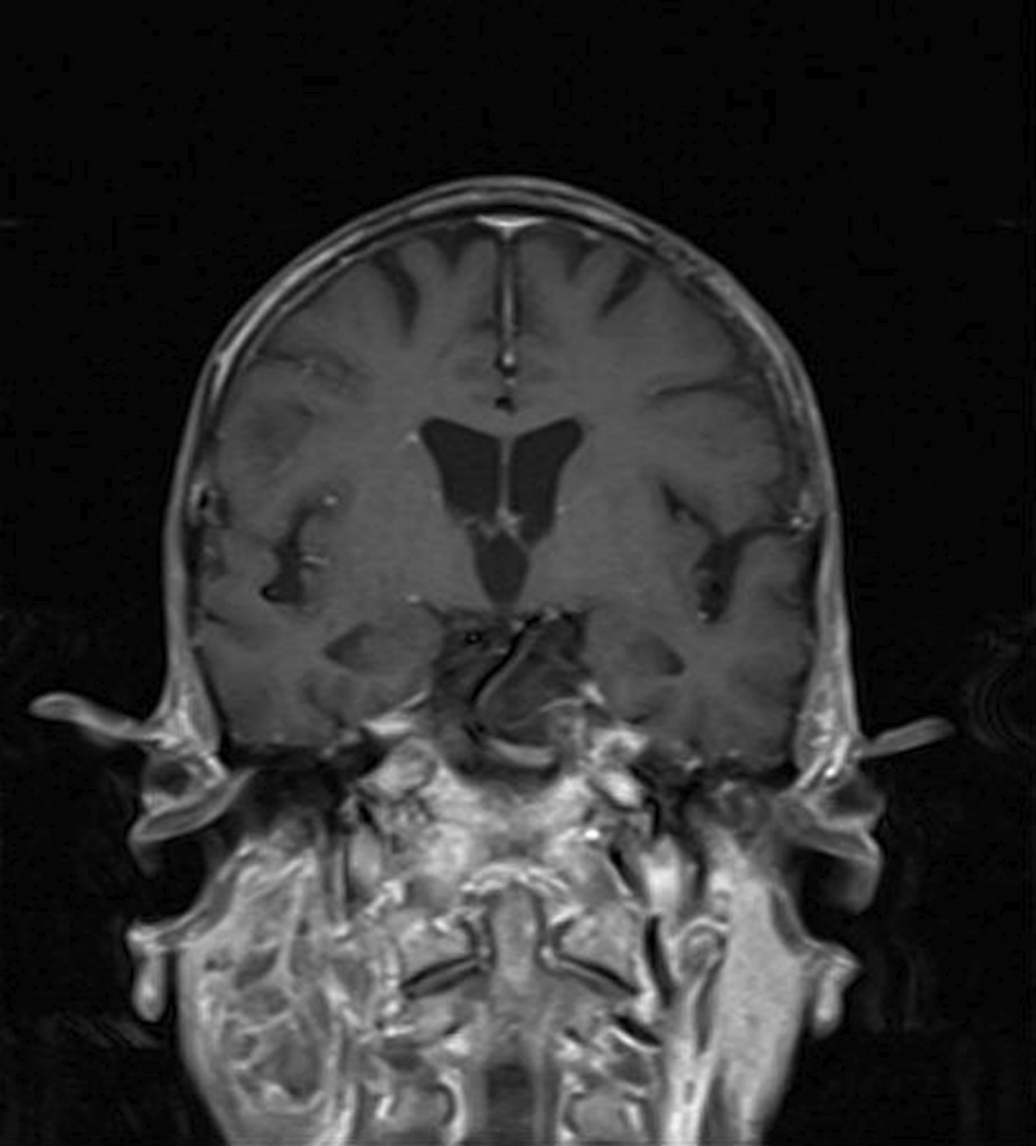
Coronal T1W brain MRI with contrast shows heterogeneous mass legions on the right side, suggesting metastatic LAP

**FIGURE 8:**
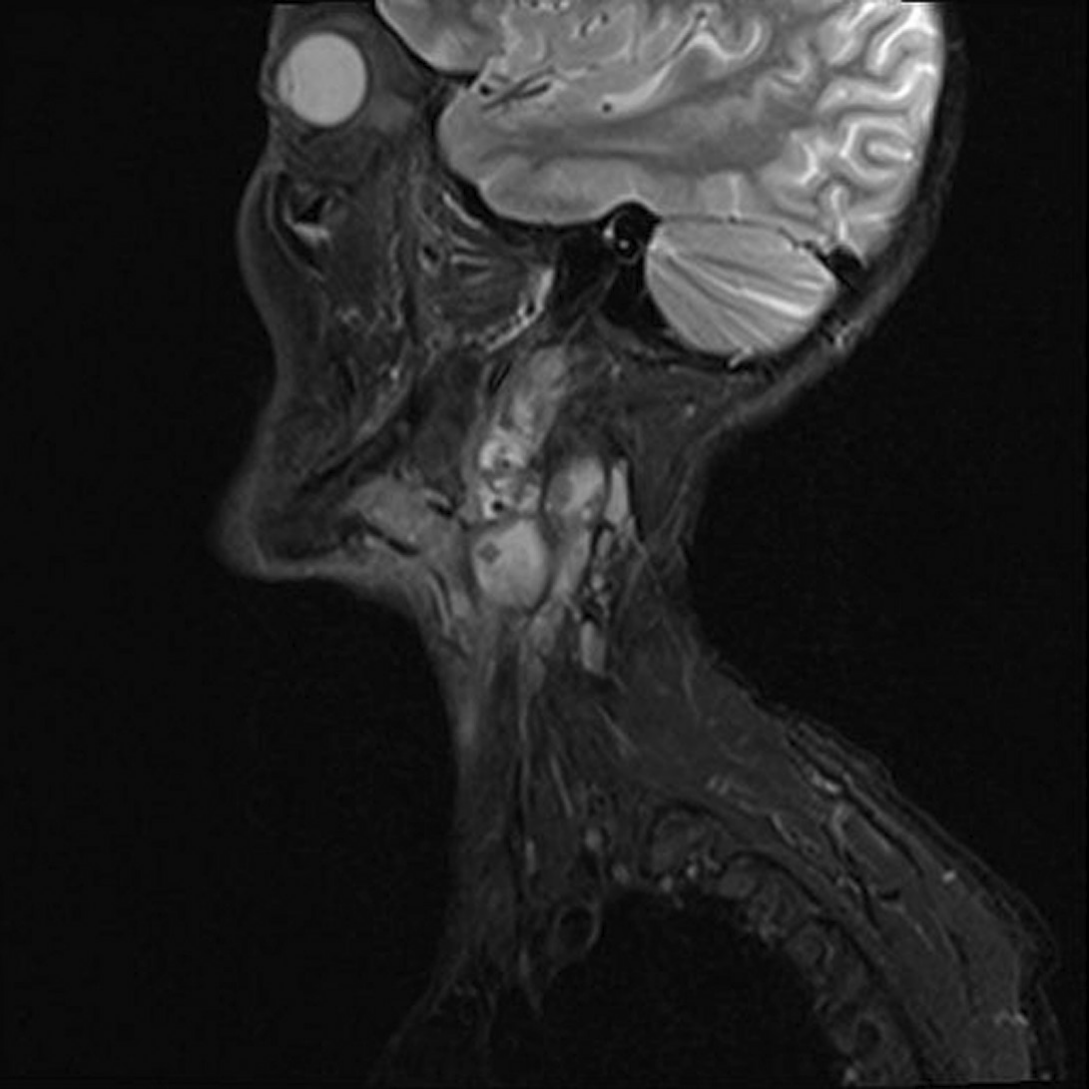
Sagittal T2W brain MRI shows heterogeneous mass legions at IIa, IIb, and III levels on the right side, suggesting metastatic LAP

The primary brain CT scan reported no bony lesion. However, 4 months later, the second brain CT performed showed osteolytic lesions in the posterior left mastoid bone and the left occipital bone without the involvement of intracranial tissues. There was evidence of calvarial bone metastasis. A contrast CT scan of the chest showed multiple nodules on both sides (up to 6 mm), indicating pulmonary metastasis. However, the contrast CT scan of the abdomen was completely normal. In MRI findings, the scalp had significantly irregularly thickening over the left posterolateral aspect of the posterior fossa, associated with bone destruction of the medial part of the left occiput with intracranial extra‐axial extension, consistent with a malignant process such as sarcoma. Notably, the cerebellar hemisphere appeared intact, and no bony lesion was detectable (Figure 6). Furthermore, bilateral well‐demarcated mass lesions dorsal to submandibular glands at levels of IIa, IIb, and III on the right side and levels of IIa and IIb on the left side were observed, which have intermediated signal intensity on T1W, hyper signal intensity on T2W sequences, and heterogeneous enhancement following administration of contrast, suggesting of metastatic lymphadenopathy (Figures 7 and 8). The rest of the physical examinations, the laboratory findings, and the cardiac SPECT scan were also normal.

**FIGURE 9:**
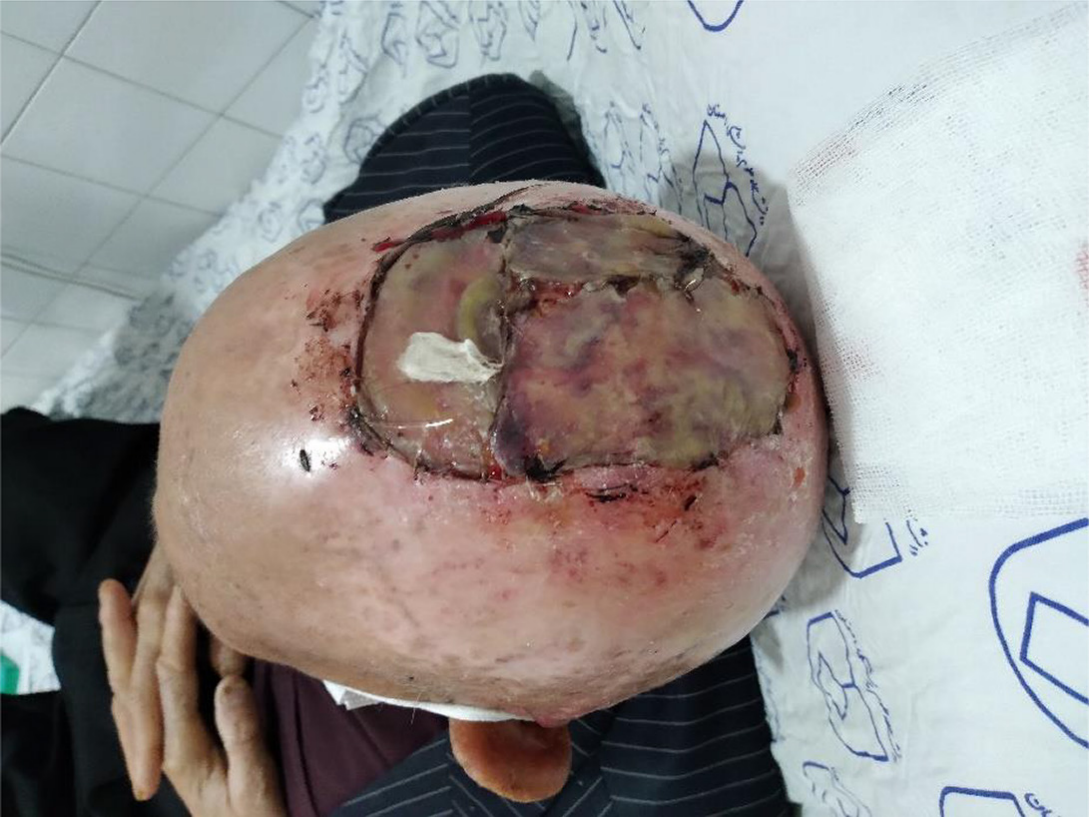
Reconstruction surgery was performed following the resection of the SCC tumor

According to his sarcoma's unresectable condition, the patient underwent chemotherapy with the AIM regimen, including doxorubicin (Adriamycin), ifosfamide, and mesna. The patient received six cycles with a 21 days interval. However, he did not respond well. Hence, the oncologist started salvage therapy with GemTaxol (Paclitaxel and gemcitabine). After the third cycle of salvage therapy, the SCC was resected entirely, and the skin grafts from his thigh were applied (Figure 9). Unfortunately, the skin grafts failed after the fourth cycle of salvage therapy. Subsequently, palliative care was considered due to the high risk of infection.

## 3 DISCUSSION

In 1902, 7 years after the X‐ray was discovered, Frieben reported the first case of radiotherapy‐induced skin malignancy. Nowadays, one of the most concerning long‐term effects of ionizing radiation is the induction of malignant disease.^2^ In the last century, radiotherapy was frequently used to treat benign skin diseases, including eczema, acne, psoriasis, and particularly tinea capitis. From the 1930s to the 1960s, radiation was the conventional treatment for tinea capitis, and it rescued many people. However, a small percentage of them have confronted radio‐induced skin cancers. Various studies have shown the association between radiation exposure and later increased risk of skin malignancies, especially in areas exposed, primarily the thyroid, head, and neck. Although radio‐induced skin tumors typically occur in exposed areas, such as the face or scalp, their incidence in hidden zones raises concerns about their etiology.^3^, ^5^

Moreover, Radiation dermatitis is one of the most prevalent side effects of radiotherapy, developing in up to 95% of irradiated patients. Radio‐induced scalp cancer commonly occurs on radiodermatitis and in ulcerated and scleroatrophic skin; the chance of malignant transformation is relatively high. Even with a normal‐looking scalp, a person with a history of childhood radiation has a greater incidence of cancers than the overall population.^1^, ^3^, ^6^ Thus, we should be more suspicious of these cases and sift through them. Several factors, including the nature of irradiated tissue, the total dose of irradiation, and low age at the time of radiotherapy, appear to influence the risk of radio‐induced malignancy.^3^, ^5^ Evidence exists to show that many generations after radiation, irradiated cells develop genetic instability. According to cytogenetic studies, the most specified aberration in radio‐induced cancers is a recessive mutation of tumor suppressor genes such as p53 genes.^3^, ^7^

Radio‐induced skin tumors can develop during the radiotherapy or in the weeks or months afterward but usually occur years later. The most prevalent non‐melanoma skin cancers described following radiotherapy are BCCs, SCCs, and sarcomas. BCC is the most frequent radio‐induced scalp cancer. SCCs are less common, most frequently observed after radiation for tinea capitis treatment, with a 20‐year latency time.^1^, ^2^ The third most prevalent group of radio‐induced malignancies are sarcomas, with an 11‐year latency time (3–36 years). Undifferentiated pleomorphic sarcoma is one of the most prevalent subtypes of RIS and predominantly occurs on the actinically damaged skin of elderly patients. Undifferentiated pleomorphic sarcoma mainly presents as single, firm, brownish to erythematous nodules that may invade subcutaneous structures.^8^, ^9^ However, it is a diagnosis of exclusion, defined after ruling out another important differential diagnosis, including invasive melanoma, SCC, leiomyosarcoma, liposarcoma, and metaplastic carcinoma. Hence, immunohistochemistry analysis is required to reach the final diagnosis.^7^, ^10^ In this study, according to IHC staining, the Ki67 index was about 80–90%, and the only positive marker was Vimentin. As a result, the patient was diagnosed with high‐grade undifferentiated sarcoma. RISs are reported to have a poor prognosis compared with sporadic soft tissue sarcomas, independent of tumor features and the patient's condition. The 5‐year survival of patients with RIS is 27% to 48%. It is estimated that up to 10% of cases develop regional metastasis.^4^, ^7^ Thus, complete excision with a wide surgical margin (at least 1–2 cm) is the best way of treatment and may improve the prognosis.^7^, ^8^

Nevertheless, in our case, regarding the patient's multiple metastases (i.e., metastatic lymphadenopathy, bone, and pulmonary metastasis), he was not a candidate for surgical therapy. Thus, he received chemotherapy courses with the AIM regimen. After multiple courses of chemotherapy, the SCC was resected, and skin grafts were applied. Unfortunately, that was not successful, and skin grafts were rejected. Subsequently, palliative care was considered for the patient.

## 4 CONCLUSION

In this article, we reported a case of severe chronic radiodermatitis who was diagnosed with two distinct scalp cancer. The simultaneous development of SCC and metastatic undifferentiated pleomorphic sarcoma is very rare, and to the best of our knowledge, no similar case has been reported so far. Although most radiation‐induced skin cancers are uncommon, clinicians should be aware of these long‐term complications in patients who have undergone radiotherapy. Furthermore, since most of these malignant tumors appear years after radiotherapy and their prevalence is low, we recommend that patients be educated on how to self‐control their skin and see a specialist immediately if they notice any alarm signs.

## AUTHOR CONTRIBUTIONS

Zahra Malakoutikhah involved in investigation and writing—original draft preparation. Fatemeh Mohaghegh involved in conceptualization, writing—review and editing, and supervision. Maryam Derakhshan involved in visualization, data curation, and laboratory testing. Mohammad Javad Mehdizadeh involved in visualization and data curation.

## CONFLICT OF INTEREST

None.

## ETHICAL APPROVAL

The ethical committee of Isfahan University of Medical Sciences approved the current study.

## CONSENT

Written informed consent was obtained from the patient to publish this report in accordance with the journal's patient consent policy.

## Data Availability

The authors confirm that the data supporting the findings of the current study are available within the article [and/or] its supplementary materials.
